# The Treatment of Cement Dermatitis

**Published:** 1909-05-15

**Authors:** 


### Dermatology.

### THE TREATMENT OF CEMENT DERMATITIS.

"Cement Itch," is a well-known industrial complaint, to which labourers, plasterers, and others who may have occasion to handle cement are liable. It is an acute vesiculating dermatitis due to irritation of the skin by particles of cement powder. Some skins are more sensitive than others, so that several labourers may be working with the same cement and yet only one of them may suffer. On the other hand, there are different sorts of cement, some, doubtless, more irritating than others. The lesion is very like an acute eczema, but it is readily distinguished from eczema proper; first by the distribution—the hands and the arms up to the elbow are most liable to the lesion, the chest less so, and the covered parts least of all; secondly, by the history of the patient's occupation; and thirdly, by the healing of the dermatitis when cement work is stopped, and its liability to recur whenever work is recommenced.

The treatment of the acute dermatitis is essentially by cessation of the work, together with the use of bland application such as a liniment of calamine applied upon lint, kept on with a bandage, and changed two or three times a day. Prophylaxis is, however, the thing to be aimed at. To prevent the trouble some workmen use grease or oil, applied to the skin before they begin work. Others try to use gloves, though these are seldom satisfactory, because they wear through so quickly. The best and simplest preventive is to advise the patient to wash the exposed parts carefully as soon as work is done, particular care being taken to clean the nails so that no cement is left adhering to them.

